# Secondary Mania induced by TNF‐α inhibitors: A systematic review

**DOI:** 10.1111/pcn.13302

**Published:** 2021-11-02

**Authors:** Alessandro Miola, Veronica Dal Porto, Nicola Meda, Giulia Perini, Marco Solmi, Fabio Sambataro

**Affiliations:** ^1^ Department of Neuroscience University of Padova Padova Italy; ^2^ Padova Neuroscience Center, University of Padova Padova Italy; ^3^ Casa di Cura Parco dei Tigli Padova Italy; ^4^ Department of Medicine University of Padova Padova Italy; ^5^ Department of Psychiatry University of Ottawa, Ottawa ON Canada; ^6^ Department of Mental Health The Ottawa Hospital, Ottawa ON Canada; ^7^ Clinical Epidemiology Program, Ottawa Hospital Research Institute (OHRI) University of Ottawa, Ottawa ON Canada

**Keywords:** bipolar disorders, disease‐modifying antirheumatic drugs, immune system, manic switch, TNF inhibitors

## Abstract

A growing number of studies support a bidirectional relationship between inflammation and bipolar disorders. Tumor necrosis factor‐α (TNF‐α) inhibitors have recently attracted interest as potential therapeutic compounds for treating depressive symptoms, but the risk for triggering mood switches in patients with or without bipolar disorders remains controversial. Thus, we conducted a systematic review to study the anti‐TNF‐α medication‐induced manic or hypomanic episodes. PubMed, Scopus, Medline, and Embase databases were screened for a comprehensive literature search from inception until November 2020, using The Preferred Reporting Items for Systematic Reviews and Meta‐Analyses guidelines. Out of the initial 75 references, the screening resulted in the inclusion of four case reports (each describing one patient) and a cohort study (in which 40 patients out of 7600–0.53% – experienced elated mood episodes after infliximab administration). Of these 44 patients, 97.7% experienced a manic episode and 2.3% hypomania. 93.2% of patients had no history of psychiatric disorder or psychotropic treatment. Only 6.8% had a history of psychiatric disorders with the affective spectrum (4.6% dysthymia and 2.3% bipolar disorder). The time of onset of manic or hypomanic symptoms varied across TNF‐α inhibitors with an early onset for Infliximab and a later onset for Adalimumab and Etanercept. These findings suggest that medications targeting the TNF‐α pathway may trigger a manic episode in patients with or without affective disorders. However, prospective studies are needed to evaluate the relative risk of such side effects and identify the population susceptible to secondary mania.

A growing number of studies supports a bidirectional relationship between inflammation and bipolar disorders (BDs)^1^, ^2^, ^3^: elevated levels of inflammatory markers – such as Interleukin‐1β, soluble Interleukin‐2‐Receptor (sIL2‐R), Interleukin‐6 (IL‐6) – have been reported in patients with bipolar disorders (BDs).^4^ Furthermore, serum levels of tumor necrosis factor‐alpha (TNF‐α), a cytokine also regulating synaptic function^5^ and neuronal survival,^6^ have been reported to be altered during manic,^7^ depressed,^8^, ^9^ or euthymic phases^7^ of mood cycles. In addition to the association between inflammatory markers and mood polarity, inflammation has been shown to play a central role in contributing to the neuroprogression of bipolar disorders,^1^, ^10^, ^11^ and a positive association between cytokine levels and manic symptoms severity has been reported.^12^

Moreover, lithium therapy – the mainstay treatment for BDs treatment – yields immunomodulatory effects^13^: it has been shown that successful treatment with this medication leads to the normalization of altered cytokine levels,^14^ and patients who do not respond to lithium therapy also have persistently high TNF‐α serum levels,^15^ whereas those who benefit from lithium therapy, besides retaining elevated TNF‐α levels, show an increase in anti‐inflammatory cytokines (i.e., IL‐4) levels.^16^

Given the case for the role of inflammation and innate immunity in BDs pathophysiology, molecules targeting the TNF‐α pathway have recently attracted interest as potential therapeutic compounds. Disease‐modifying antirheumatic drugs (DMARDs), including TNF‐α inhibitors (i.e., infliximab, adalimumab, certolizumab, and etanercept), have shown positive effects on the affective, cognitive, and somatic function of patients with inflammatory illnesses.^17^ In particular, the administration of TNF‐α inhibitors improves depressive symptoms in patients with psoriasis^18^ or Crohn's disease^19^ and reduces fatigue in patients with advanced cancer.^20^ However, very little is known about the safety and efficacy of these drugs in patients with BDs. Analogously to other antidepressant treatments, DMARDs may contribute to triggering a manic switch in patients with BDs. However, sparse and limited evidence on this topic has been reported. Thus, this systematic review aimed to summarize current evidence supporting the role of TNF‐α antagonists in inducing secondary manic episodes or exacerbating a mood switch in patients with or without mood disorders.

## Methods

### Protocol and search strategy

This systematic review followed a pre‐defined protocol available online (https://osf.io/mt7jb/quickfiles) and adhered to the procedures of Preferred Reporting Items for Systematic Reviews and Meta‐Analyses (PRISMA) statement^21^ (see Supplementary materials for details). A comprehensive literature search was performed in PubMed, Scopus, Medline, and Embase databases, with the following keywords: (‘infliximab’ OR ‘adalimumab’ OR ‘etanercept’ OR ‘certolizumab’ OR ‘anti‐TNF’ OR ‘TNF antagonist’ OR ‘TNF inhibitors’) AND (‘mania’ OR ‘manic’ OR ‘hypomania’ OR ‘hypomanic’). Moreover, the reference lists of included papers were screened by snowball search.

### Eligibility

Case–control, experimental, cross‐sectional, and prospective studies were considered eligible. Studies were included if (i) reported the use of TNF‐α inhibitors in patients with or without mood disorders according to the Diagnostic and Statistical Manual (DSM) or the International Classification of Diseases (ICD); (ii) a qualitative measure of manic or hypomanic episodes induced by TNF‐α inhibitors as side effects; (iii) were written in English. Commentaries, editorials, and reviews were excluded. All articles published until November 2020 were included, while no publication status restrictions were imposed.

### Data extraction

Every reference was screened by at least two researchers independently (A.M. and V.D.P.), any disagreement was discussed between the two, and whenever it was not possible to make a decision, a third researcher was involved in the discussion (F.S.). Once the full‐text articles were selected, the data retrieved have been entered into a spreadsheet. Sample size, demographics, previous treatment, treatment response, adverse effects, and follow‐up time were extracted. The analysis of the data was made by comparison. The heterogeneity of the results, such as the type of studies identified, did not allow us to perform a meta‐analysis. A narrative synthesis was considered the best approach to describe and analyze the results.

## Results

**Fig 1:**
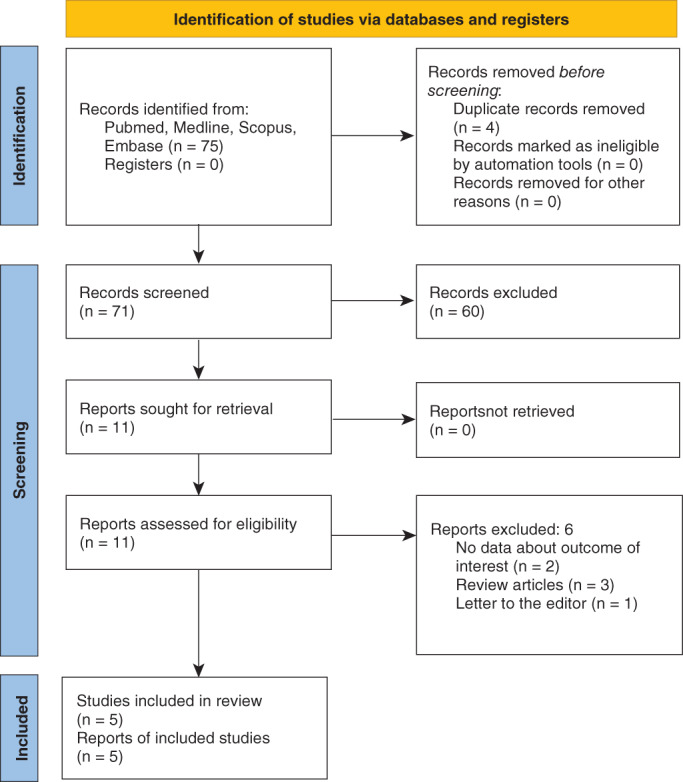
PRISMA study flow chart.

The database search, after duplicates removal, brought a total of 71 records. Following the inclusion/exclusion criteria, the screening resulted in the inclusion of 5 full‐text articles (Fig. 1). The evidence available regarding the manic or hypomanic induced by TNF‐α antagonists is limited to four case reports^17^, ^22^, ^23^, ^24^ and a cohort study.^25^ Information about the patients, such as demographics, previous treatment, treatment response, manic or hypomanic adverse effects, and follow‐up time, were extracted and summarized in Table 1. A total of 44 patients experienced manic or hypomanic episodes after treatment with a TNF‐ α antagonist (n = 1, etanercept; n = 1, adalimumab; n = 42, infliximab). In particular, 97.7% showed an induced manic episode, and just 2.3% of patients (n = 1) experienced hypomania. Several rheumatological illnesses were associated with anti‐inflammatory‐induced hypomanic or manic episodes: psoriasis,^25^ psoriatic arthritis,^24^ ankylosing spondylitis,^17^, ^23^, ^25^ Crohn's disease,^22^, ^25^ and ulcerative colitis.^25^ Most patients had no history of psychiatric disorder or psychotropic treatment (93.2%), and only 6.8% (n = 3) had a previous psychiatric disorder (dysthymia, n = 2^17^, ^23^; bipolar disorder, n = 1^24^). The onset of manic or hypomanic symptoms differed across TNF‐α inhibitors: with an early onset for Infliximab (after first administration) and a later onset for Adalimumab and Etanercept (after second administration). No cases of secondary mania or hypomania after certolizumab administration have been reported so far.

**Table 1:** Characteristics of included studies

Author, year	Study design	Sample size	Age (Years), sex	Previous episodes	Primary diagnosis	TNF‐α antagonist	Psychiatric medication	Time of hypomania/ mania onset	Hypomania/mania Treatment
Kaufman K.R., 2005^24^	Case report	1	21, F	Atypical depression and manic sertraline‐ induced episode	Psoriatic arthritis	Etanercept (25 mg s.c./ 2 weeks)	Lamotrigine (37.5 mg q.h.s)	Mania after the 2nd administration	Stop Etanercept + starting Valproate (1000 mg/day), + oxcarbazepine (1200 mg/day), and ziprasidone (80 mg/day), clonazepam (1 mg/day)
Brietzke E. & Lafer B., 2010^23^	Case report	1	43, F	Dysthymia	Ulcerative colitis	Infliximab (dosage not available)	Citalopram (40 mg/day)	Mania after the 1th administration	Stop infliximab
Austin M., 2012^22^	Case report	1	62, M	none	Crohn's disease	Infliximab (dosage not available)	none	Mania after the 1st administration	Starting Olanzapine (5 mg/day)
Ghosshoub E. *et al*., 2016^17^	Case report	1	25, M	Dysthymia	Ankylosing spondylitis	Adalimumab (40 mg s.c. / 2 weeks)	None; escitalopram (dosage not available) for one month	Hypomanic symptoms after the 2nd administration; manic symptoms after starting antidepressant	Stop Adalimumab and starting + valproate (750 mg/day) and aripiprazole (10 mg/day)
Thillard E‐M *et al*., 2020 ^25^	Historical cohort study	40 patients, of 7600 treated with infliximab	n.a., 47,7% M	none	Rheumatoid arthritis, psoriasis, ulcerative colitis, Crohn's disease, ankylosing spondylitis	Infliximab	none	Mania after 5 days (median time interval)	n.a.

M, male; F, female; q.h.s, quaque hora somni; S.c., subcutaneously.

## Discussion

To our knowledge, this is the first systematic review conducted to date assessing the available evidence about the role of TNF‐α antagonists in inducing secondary manic episodes or exacerbating a mood switch in patients with or without mood disorders.

Hitherto, a cohort study,^25^ two clinical cases for infliximab,^22^, ^23^ one for etanercept,^24^ and one for adalimumab^17^ support the case for these drugs of inducing manic episodes in patients receiving treatment for inflammatory illnesses, especially for patients without a history of bipolar disorders or hypomanic/manic symptoms. No cases of secondary manic or hypomanic episodes after treatment with certolizumab have been reported so far.

TNF‐α antagonists had recently attracted interest as potential therapeutic compounds for mood disorders.^6^ However, few studies have been published regarding their safety and efficacy for treating patients with mental disorders or patients with inflammatory diseases and comorbid psychiatric disorders. In this systematic review, we sought to analyze the available evidence on the putative role of TNF‐α in triggering hypomanic or manic episodes in patients with or without a mental disorder. The vast majority of patients who experienced a manic/hypomanic episode (93.2%) had no history of psychiatric disorders until exposure to TNF‐α inhibitors. Except for the case reported in the study by Ghossoub and colleagues (Table 1), only manic episodes have been reported to be triggered by Infliximab, Adalimumab, or Etanercept. This is in line with previous evidence that supports the case for the role of the immune system response in the onset and clinical presentation of bipolar disorders.^26^ Furthermore, it has been reported that the thymic phases of the disorder (depression, mania/hypomania, and euthymia) show different cytokine profiles, suggesting an association between inflammatory dysfunction, mood state, and mood phase.^3^, ^7^, ^12^ In patients experiencing a manic episode, pro‐inflammatory cytokines (e.g., TNF‐α, IL‐1, IL‐6), soluble receptors of IL‐2, soluble TNF‐α receptor type 1 (sIL‐2R and sTNFR1, respectively), and C reactive protein (CRP) are generally increased when compared to a control population.^1^, ^2^, ^3^ The association between inflammation and depressive episodes in both bipolar and unipolar depression is supported by several studies.^27^, ^28^ Similarly to manic episodes, serum levels of many inflammatory markers (CRP, TNF‐α, IL‐6, IL‐1β, sTNFR1, and CXCL10) are elevated during depressive episodes,^29^ and this alteration correlates with increased depression severity.^30^ Euthymia is generally associated with normal cytokine levels, except for sTNFR1, which remains elevated during partial or complete remission.^8^, ^31^, ^32^ A systematic review of cytokine profiles in patients with bipolar disorder suggested that several cytokines (e.g., sIL‐2R, IL‐6) are “state‐related” markers in medication‐free bipolar disorder.^13^ On this cytokine background, a pro‐inflammatory response (marked by an increase of, for example, TNF‐α and CRP levels) might distinguish, on serum, a manic episode from euthymia. In other words, some cytokines or clusters of cytokines might be altered independently of the mood phase, while other pro‐inflammatory molecules elevate specifically during manic or hypomanic episodes.

A recent systematic review and meta‐analysis finally confirmed altered peripheral markers in BD, according to which IL‐6 seems to be a trait marker for BDs, while CRP and TNF‐α could constitute state markers, as they are increased during mood episodes,^33^ a feature that could also represent a fruitful entry‐point for the prevention of suicide attempts.^34^

Increased cytokine variability suggests that a subset but not all patients may exhibit cytokine elevations as part of a manic episode.^33^

**Fig 2:**
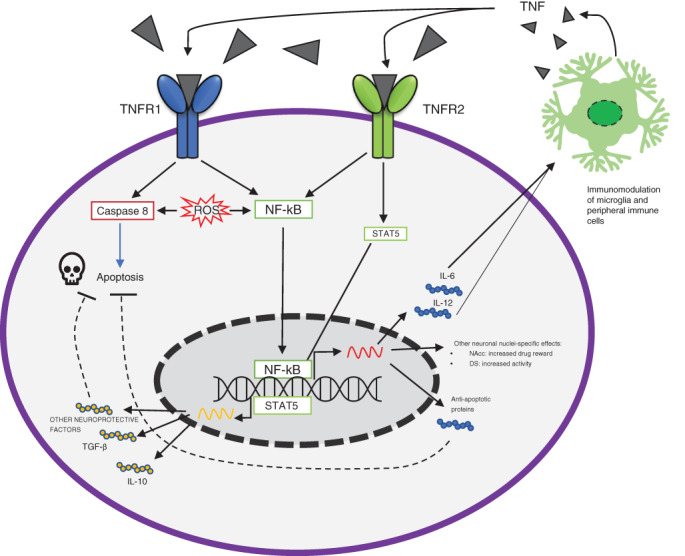
TNF‐α mediated pathways are involved in a delicate balance between cell death and inflammation. TNFR, tumor necrosis factor receptor; STAT5, Signal transducer and activator of transcription 5; TGF, transforming growth factor; IL, interleukin; NF‐kB, nuclear factor kappa‐light‐chain‐enhancer of activated B cells; NAcc, nucleus accumbens; DS, dorsal striatum. TNF‐α exerts pleiotropic effects on neurons and neighboring cells in the central nervous system. TNF‐α, through the binding of TNFR‐1, initiates two multiple‐step cascades: an apoptotic one (Caspases‐mediated) and a pro‐survival one (NF‐kB‐mediated). The balance between the two signaling pathways is also regulated by ROS levels.^36^ The TNFR‐2‐mediated cascade leads to the activation of NF‐kB and STAT5, which eventually leads to the transcription of immuno‐modulatory genes (e.g., genes encoding IL‐6, IL‐10, IL‐12) and the production of neuroprotective molecules. These factors are released from the neurons and affect the neighboring microglia, eventually regulating the production and release of TNF‐α, thus closing an immuno‐modulatory loop between neurons and neighbor immune cells. Furthermore, TNF‐α exerts neuronal nuclei‐specific effects: for example, TNF‐α enhances drug reward responses through regulating NAcc neurons, whereas through the dorsal striatum neurons, it mediates an increase in locomotor activity.^5^

TNF‐α is a major Th1‐class pro‐inflammatory cytokine, that can bind to TNFR1 and/or TNFR2, activating downstream signaling pathways that mediate a wide variety of biological responses, including apoptosis, cell differentiation, proliferation, survival, homeostatic synaptic plasticity and inflammation^5^, ^35^, ^36^, ^37^, ^38^ (Fig. 2). The gene encoding TNF‐α is located on chromosome 6, which has been reported to be a genetic Major Depressive disorder‐susceptibility region.^39^, ^40^ Nonetheless, a recent systematic review and meta‐analysis revealed that neither the genotypes of the TNF‐α G308A gene nor allele frequencies might represent an independent risk factor of depression.^41^

TNF‐α contributes to brain development, particularly by modulating hippocampal growth and function.^42^, ^43^ However, in several disorders, increased levels of this cytokine activate microglia, which then leads to demyelination and/or neuronal degeneration.^44^, ^45^, ^46^ Furthermore, stimulated microglia causes an increase in cytotoxic molecules, including TNF‐α, which is regulated by a positive feedback mechanism of autocrine activation.^43^, ^45^, ^47^ Moreover, cytokines including TNF‐α can modulate neural activity and neurotransmitter systems. Chronic exposure to high levels of inflammatory cytokines and central neurotransmitters impairment may play a role in psychiatric disorders, including bipolar and mood disorders.^48^, ^49^ The activation of inflammatory signaling pathways underlying cytokine behavioral effects results in changes in monoamine and neuropeptide systems, chronic HPA axis activation, purinergic system abnormalities, increases oxidative stress and glutamate excitotoxicity, as well as decreases in growth factors, such as brain‐derived neurotrophic factor (BDNF).^3^, ^27^, ^49^, ^50^, ^51^ In addition, inflammatory cytokines inhibit neurogenesis through the activation of nuclear factor B^52^ and induce cognitive impairment and sleep alterations *via* the tumor necrosis factor‐α pathway that are crucial factors in the pathogenesis of BDs.^53^, ^54^

From the therapeutic point of view, given the role of the interaction between the Serotonin/Norepineprhin system and Th1/Th2 immune response balance,^55^ treatments inhibiting inflammatory cytokines have been tested for treatment‐resistant depression (TRD).^56^ The randomized controlled trial (RCT) by Raison *et al*. assessed infliximab safety and efficacy for patients with treatment‐resistant unipolar and bipolar depression. Although the overall antidepressant effect was negative, a significant antidepressant effect was observed in the subgroup with elevated serum C reactive protein levels.^56^ The result of this trial supports the idea of inflammatory biotypes as if individuals with a mood disorder that are exhibiting pro‐inflammatory balance^26^ would be more likely to benefit from an anti‐inflammatory treatment. A more recent RCT assessed the efficacy of infliximab in treatment‐resistant bipolar depression, and it included patients with a biochemical and/or phenotypic marker of the inflammatory response.^26^ This RCT failed to show a significant reduction of depressive symptoms in the treatment arm with respect to the placebo arm, except for a subgroup of patients who reported a history of childhood physical and/or sexual abuse (an anamnestic event that can contribute to a pro‐inflammatory state in adulthood^57^). However, recent evidence highlights that amelioration of anhedonia symptoms by infliximab administration can be predicted on the basis of inflammatory biotypes.^58^ Taking together the evidence presented above, we propose that the role of TNF‐α elevation in patients with bipolar disorder may be secondary, or even compensatory, to the onset of the manic phase. That is, the manic episode precedes the TNF‐α elevation. We suggest this interpretation based on three findings: (i) low serum concentration of TNF‐α is associated with sustained remission, but not with the immediate remission phase.^12^ This might point that the reduction of this cytokine level lags with respect to mood symptoms reduction. Thus, it can be inferred that changes in manic symptoms temporally precede the alterations of this inflammatory marker instead of the other way around. Moreover, (ii) a rapid reduction of TNF‐α levels (i.e., after TNF‐α inhibitors treatment) might be a trigger of manic switches in a subgroup of patients, as described in this review. This phenomenon suggests that higher levels of TNF‐α somehow might act as a “brake” (a compensation, indeed) to the manic episode, and the rapid inhibition of TNF‐α activity exacerbates a manic phase. Further evidence supporting an anti‐manic role of high levels of TNF‐α levels come from medication‐free patients (iii): the normalization of TNF‐α levels during euthymic phase is observed only in medication‐free patients (at higher risk of recurrence of manic/hypomanic episode), but not in patients who benefit from taking lithium monotherapy for manic episode prevention, for whom also an increase of anti‐inflammatory cytokines (IL‐4) is reported.^16^ However, we underline that this consideration is practical only as a working hypothesis for further longitudinal studies, which can better elucidate the temporal relationship between cytokine levels and mood episodes. Lastly, we wish to underline that the risk of DMARDs‐induced mania or hypomania (0.53%, according to^25^) is 10 times lower when compared to the incidence of these adverse events due to other anti‐inflammatory treatments, e.g., corticosteroid exposure (above 5%, depending on the steroid dosage and illness treated^59^). In general, the risk of secondary mania following anti‐inflammatory drug administration is much lower compared with antidepressant‐induced mania (approximately 18%, as reported by^60^). This evidence is not surprising since patients taking antidepressant medications are usually diagnosed with a psychiatric disorder (most likely a mood disorder) and, therefore, more amenable to a manic switch. The population taking TNF‐α inhibitors included in this review, instead, had no prior history of mental disorders.

Some limitations to this systematic review have to be acknowledged. First, the available evidence is limited to four case reports and a cohort study, all characterized by small sample sizes. Second, given the lack of prospective evidence, we cannot draw firm conclusions on causality. Third, the population who experienced secondary mania is heterogeneous both in terms of diagnosis and treatment.^25^ In fact, the definitions of manic switch and the timeframes of the emergence of the affective episodes after treatment with TNF‐α inhibitors vary among the reported literature. Moreover, the mood polarity before treatment initiation could not be assessed, thus hampering the possibility to clearly define a treatment‐emergent affective switch.^61^ Given the scarcity and low level of evidence of the literature published so far, the incidence of secondary manic or hypomanic episodes cannot be assessed with reliable confidence, and those who experienced invalidating mood episodes might be a small subgroup of patients that would otherwise benefit from DMARDs therapeutic regimens.^62^, ^63^, ^64^, ^65^

In conclusion, these psychiatric adverse events' characterization is essential for adequately assessing the risk–benefit ratio and improving the management of these events when they occur. Prospective studies with a multidisciplinary approach, close psychiatric monitoring, and serial cytokine levels measures are warranted to demonstrate the relative risk of secondary mania after TNF‐α inhibitors administration, or abrupt discontinuation (as seldom reported for SSRIs^66^), and to further elucidate the relationship between mood phases and peripheral inflammatory marker alterations.

## Disclosure statement

The authors declare no conflict of interest.

## Author contributions

A.M., V.D.P., and F.S. conducted the literature review. F.S., G.P., and M.S. guided the areas for discussion. A.M., V.D.P., N.M., and F.S. conducted the statistical analysis. A.M., V.D.P., and N.M. wrote the draft of the manuscript. All authors have equally contributed to the critical revision of the manuscript.

## Funding

The authors received no specific funding for this work.

## Supporting information


**Appendix S1**. Supporting information.
